# A Retrospective Analysis of Breast Cancer Presentation Among Young and Older Women in an Indian Cohort of 70 Patients

**DOI:** 10.7759/cureus.61239

**Published:** 2024-05-28

**Authors:** Neelesh Shrivastava

**Affiliations:** 1 Department of Surgical Oncology, All India Institute of Medical Sciences Bhopal, Bhopal, IND

**Keywords:** fine needle aspiration cytology (fnac), modified radical mastectomy (mrm), neoadjuvant chemotherapy (nact), american joint cancer committee (ajcc), infiltrating ductal carcinoma (idc), ductal carcinoma in situ (dcis)

## Abstract

Introduction

In females, carcinoma of the breast is a common malignancy. Disease management can be challenging for the treating clinician if the condition is presented in a locally advanced stage. Clinical presentation of a disease in a defined area provides a comprehensive map to target the at-risk population efficiently and select the appropriate intervention accordingly. In this retrospective study, we analyzed different factors that can affect breast carcinoma outcomes by assessing patients for a specific period of one year.

Methods

This is a retrospective study of carcinoma of breast patients and was conducted between 2017 and 2018.

Results

We reported a 25.83% incidence of breast cancer during the study period. A painful breast lump was present in 54.2% of patients, axillary nodes were present in 50% of patients, ulcers were present in 10% of patients, and nipple discharge was present in 8.5% of patients. According to the side and quadrant of involvement, the right side was the most common site of involvement in 55.7% of patients, and the upper outer quadrant was the most common quadrant involved in 61.4% of patients. The most familiar stage of the presentation was stage II, presented in 45.7% of patients. The most common histology was infiltrating ductal carcinoma, presented in 85.7% of patients.

Conclusions

This retrospective cohort study shows that carcinoma of the breast is a predominant malignancy among middle-aged females in developing countries such as India. This predominance is due to unawareness regarding disease symptoms and the fear of diagnosed malignancy during the investigation of symptoms. Early detection by screening and intervention at an early stage is the core of treatment success in this malignant disease. However, it is still challenging to apply screening as a tool to pick up early malignant disease in developing countries like India.

## Introduction

Breast cancer is the most common cancer in women and a significant cause of premature mortality, with approximately 50% of cancers occurring in those under 65 years of age [1]. The “triple assessment,” which involves clinical examination, imaging (mammography), and core needle biopsy, is a gold standard in assessing patients with painless breast mass. Breast cancer-related death is attributable mainly to metastasis [2]. Among different metastatic sites, bone seems to be the most frequent site for metastasis. Several studies have indicated that breast cancer patients with bone metastasis survived longer than patients with visceral metastasis [2]. The most valuable prognostic factors in breast cancer patients are lymph node status, primary tumor size, hormone receptor status, histological type, and histological grade [3]. The clinicopathological profiles of breast cancer patients from urban and rural locations varied significantly [4]. In India, incidence rates differ considerably among metropolitan, urban, and rural areas. While the metropolitan areas of Delhi and Chennai reported age-adjusted rates of 41 and 37.9 per 100,000 population, the incidence rates in Pune and Nagpur were 26.4 and 29.3, respectively [4]. By 2026, projections indicate that the annual incidence of breast cancer will rise to 1.8 million new cases [5]. China, the USA, and India bear a third of the world's weight for breast cancer [6]. In low- and middle-income nations, breast cancer is becoming a more serious public health issue [7]. Breast cancer has quickly emerged as a significant worldwide health concern [8]. The incidence of breast cancer has been increasing worldwide over the past several decades, especially in developing countries, with the highest increase in Asian countries. Breast conservation has emerged as a promising treatment option for early-stage disease, especially T1 lesions with no axillary nodes [9].

However, in the Indian setup, treatment-seeking is delayed, especially in rural areas. Conventionally, a gap of more than three months between noticing symptoms and commencing treatment is a delay [10]. Nowadays, many patients with small-size primary require the neoadjuvant approach in breast cancer management, especially in triple-negative and HER-2 enriched variety; sentinel lymph node biopsy or axillary dissection is a choice for axillary staging in breast cancer, but axillary dissection is still the standard treatment in cases of biopsy-proven node-positive breast cancer in upfront settings. Axillary dissection is associated with the morbidity of seroma, pain paresthesia, and lymphedema. Independent predictors for seroma formation are body mass index and extent of axillary dissection [11]. Awareness programs are needed to increase awareness regarding the disease in developing countries such as India. A study was conducted to examine the knowledge, attitudes, and practices about breast cancer in rural women in developing countries, which shows that there is wide heterogeneity in breast cancer awareness programs, which are more in urban areas but deficient in remote and rural areas, and this is the reason for different clinical presentations of disease in developing countries [12]. This study aims to analyze the clinical presentation, demographic characteristics, and outcomes of breast cancer patients in an Indian cohort to identify patterns and suggest improvements in early detection and treatment.

## Materials and methods

This is a retrospective cohort hospital-based study that included all patients diagnosed with breast cancer and admitted to the institution from July 2017 to August 2018. We recorded their characteristics with the following headings: (1) incidence of breast carcinoma in the patients admitted under the department of surgical oncology during the study period among total admitted patients; (2) patient characteristics (sex, age, marital status, residence, socioeconomic status, literacy); (3) presenting complaints (lump, painful lump, axillary node, ulcer, nipple discharge, family history, associate complaints); (4) tumor characteristics (side involved, quadrant involved, clinical stage, according to the American Joint Committee on Cancer [AJCC] 8th Edition, histopathology); (5) Surgical characteristics (type of surgery, approach of chemotherapy); (6) postoperative characteristics (hospital stay, mortality).

After approval of the ethical committee, the study was started. We collected data on an electronically defined system (Google sheet) and entered them sequentially. We excluded patients admitted during the specified period with breast lumps and who did not have a definitive histopathological diagnosis of breast cancer. We also excluded patients discharged from the hospital at their request. We included patients who were operated on outside our institution and whose histopathological slides were reviewed inside the institution with breast cancer diagnosis, followed by those who had taken adjuvant chemotherapy in our institution or patients who had taken neoadjuvant chemotherapy (NACT) outside with the same histopathology as in the aforementioned review criteria of the institution and who were operated in our hospital.

We recorded and categorized data according to numerical variables (e.g., sex, marital status, residence, literacy, family history) and continuous variables (e.g., socioeconomic status, tumor stage, side, quadrant). We performed and recorded analysis with mean, median, and mode variables using SPSS (IBM Corp., Armonk, NY).

Clinical staging was done as per AJCC 8th Edition; a modified Kupuswammy scale was used for socioeconomic status, and the following three categories were made: upper class and upper middle class as high socioeconomic status, lower middle and upper lower as mid socioeconomic status, and lower class as low socioeconomic status.

For literacy, the following subjective criteria were used: a person who can read or write a language was considered literate, a person who can read but not write a language was considered illiterate, and a person who neither can read nor write was considered illiterate.

During the retrospective data collection, we contacted all patients personally by telephone. Still, many of the treated patients were not traceable; thus, we included only a limited number of patients in the study.

## Results

In this retrospective study, 70 breast carcinoma patients were included who were admitted to the surgical oncology ward from August 2018 to July 2019.

Incidence

Table 1 provides the incidence of breast cancer among the total admitted cancer patients in the surgical oncology ward. Breast cancer accounts for 25.83% of all cancer patients hospitalized.

**Table 1:** Incidence of breast cancer among admitted patients

Total no. of cancer patients	Total no. of breast cancer patients	Percentage of breast cancer among the total cancer patients
271	70	25.83%

Patient characteristics

All patients were females, and no male patients were recorded during the study. The patients varied in age from 28 to 85 years, with a mean age of 45.2 years. Table 2 demonstrates the patient characteristics and shows that 57.14% of patients were aged 50 years or younger, while 42.86% were older than 50 years. One patient (1.5%) was unmarried, while 69 (98.5%) were married. The majority of patients (67.1%) came from rural regions, while 32.8% came from metropolitan areas. Overall, 47 (67.1%) patients were from a low socioeconomic class, and 22 (31.4%) patients were from a medium socioeconomic class, whereas one (1.5%) patient was from the upper socioeconomic class. The majority of the patients were uneducated (71.4%), and 28.5% were educated.

**Table 2:** Characteristics of carcinoma of breast patients

Characteristics	Number of patients (n=70)	Frequency (%)
Sex		
Male	0	0
Female	70	100
Age (years)		
≤50	40	57.14
>50	30	42.86
Marital status		
Married	69	98.5
Unmarried	1	1.5
Residence		
Rural	47	67.1
Urban	23	32.8
Socioeconomic status		
Low	47	67.1
Middle	22	31.4
High	1	1.5
Literacy		
Illiterate	50	71.4
literate	20	21.6

Presenting complaints

Breast lumps were the most common presenting complaint in all analyzed patients. Table 3 shows the patient’s presenting complaints and demonstrates that the breast tumor was painful in 38 (54.2%) patients. In 35 (50%) patients, axillary nodes were palpable. Ulcers over the breast were seen in seven (10%) patients. Six individuals (8.5%) had nipple discharge, which was bloody and from the unilateral side. Weakness was reported in 58 (82.8%) of the patients, anorexia in 38 (54.2%), and weight loss in 30 (42.8%) of patients. One (1.5%) of the 70 patients had a family history of breast cancer and underwent breast cancer gene (BRCA) testing.

**Table 3:** Presenting complaints of carcinoma of breast patients

Characteristics	Number of patient (n=70)	Frequency (%)
Presenting complaints		
Lump	70	100
Painful lump	38	54.2
Axillary nodes	35	50
Ulcer on breast	7	10
Nipple discharge	6	8.5
Associated complaints		
Weakness	58	82.80
Anorexia	38	54.20
Weight loss	30	42.8
Family history		
Negative	69	98.5
Positive	1	1.5

Tumor characteristics

Involvement in the right breast (55.7%) was more common than in the left (42.8%). One case (1.5%) was noted to have bilateral breast involvement. Table 4 represents the tumor characteristics and demonstrates that the upper outer quadrant was most frequently affected in patients with breast cancer (61.4%), followed by the lower outer quadrant (17.1%). We recorded involvement in 4.2% of patients in the upper inner quadrant, 4.2% in the lower inner quadrant, 11.4% in more than one quadrant, and 1.5% in the central quadrant. Most of the patients with carcinoma breast presented in stage II, 32 (45.7%), followed by 23 (32.8%) in stage III, 12 (17.1%) in stage IV, and only three (4.2%) patients in stage I disease according to AJCC 7th staging. The majority of patients (85.7%) had infiltrating ductal carcinoma (IDC) on histopathological analysis, whereas 14.3% of the remaining 70 patients had ductal carcinoma in situ.

**Table 4:** Tumor characteristics of carcinoma of breast patients IDC, ductal carcinoma; DCIS, ductal carcinoma in situ

Characteristics	Number of patient (n=70)	Frequency (%)
Side		
Right	39	55.7
Left	30	42.8
Bilateral	1	1.5
Quadrant		
Upper outer	43	61.40
Lower outer	12	17.10
Upper inner	3	4.2
Lower inner	3	4.2
Central	1	1.5
>1 quadrant	8	11.4
Clinical stage		
I	3	4.2
II	32	45.7
III	23	32.8
IV	12	17.1
Histopathology		
IDC	60	85.7
DCIS	10	14.3

Surgical characteristics

All 70 patients underwent surgical intervention. Table 5 shows that the most common surgical procedure was modified radical mastectomy (MRM), accounting for 52.85% of cases, and breast conservative surgery (wide local excision with axillary dissection) was performed in 47.15% of cases.

**Table 5:** Surgical characteristics of carcinoma of breast patients MRM, modified radical mastectomy; BCS, breast conservative surgery

Characteristics	Number of patients (n=70)	Frequency (%)
Surgery		
MRM	37	52.85
BCS	33	47.15

Preoperative chemotherapy

Table 6 demonstrates that out of 70 patients, 42 patients underwent NACT because of locally advanced disease, and 28 patients underwent upfront surgery.

**Table 6:** Preoperative chemotherapy received in carcinoma of breast patients NACT, neoadjuvant chemotherapy

Characteristics	Number of patients (n=70)	Frequency (%)
Intervention		
NACT	42	60
Upfront surgery	28	40

Postoperative characteristics

It is evident from Table 7 that the duration of hospital stay was 0-5 days in 60 (85.71%) patients and more than 5 days in 10 (14.29%) patients. However, there was no postoperative mortality in all operated cohort groups.

**Table 7:** Postoperative characteristics of carcinoma of breast patients

Characteristics	Number of patient (n=70)	Frequency (%)
Hospital stay		
<5 days	60	85.71
>5 days	10	14.29
Mortality		
Admitted	0	0

## Discussion

Although lung cancer is still the most common cancer among males, breast cancer is the most common cancer in females. In recent years, increasing the patient’s chances of survival via multimodality treatment has become a significant area of research. Current trials show the definitive role of conservative surgery and limited axillary nodal dissection in decreasing postoperative morbidity related to surgical procedures. One of the most notable aspects of breast cancer is the variance in incidence and death across different countries. India is a developing country, and the incidence of breast cancer is increasing among the Indian population, especially in the urban setting, due to growing awareness and increased screening for cancer. In this retrospective study, breast cancer accounts for 25.83% of all cancer patients hospitalized in the Department of Surgical Oncology. However, other researchers did not calculate this variable. The mean age at which breast carcinomas occurred in our research was 45.2 years. This is comparable with the findings of Rathod et al., who showed the mean age of presentation as 47.2 years in their study [4]. Similarly, Pandey et al., in their research, showed that the mean age of presentation of breast cancer was 46 years [5].

Nulliparity is a well-understood risk factor for developing breast carcinoma. In our retrospective study, only one patient was nulliparous, and we found most married women to be commonly affected by breast cancer (98.5%). This finding was supported by Suhani et al., who showed in their study that the majority of married women were affected by breast cancer [6]. Nene et al. demonstrated that 83.5% of married women developed breast carcinoma [7]. Familial cancer syndrome contributed to 5% of all breast carcinoma cases. In this retrospective study, only one patient (1.5%) had a family history of breast cancer. This is supported by the study of Nene et al., who demonstrated family history in four (4.4%) out of 99 patients [7]. In this retrospective study, all patients presented with lumps of varying duration. Nene et al. demonstrated the same findings in their study, where 98 patients out of 99 presented with breast lumps [7], and 54.2% of patients had painful swelling. This is because lumps in the breast are often ignored, and when they increase or become sore, patients get worried about carcinoma and present it to the clinician. In most studies, breast cancer commonly involves the left side more than the right side. In our retrospective study, the right breast was involved in 55.7% of patients; this is supported by Augustine et al., who found that the right side was more commonly affected than the left side (54.4% versus 45.6%) [9]. In contrast, Doval et al. reported that the left side (51%) was more common than the right side (49%) [8]. The involvement of the side of the breast is a subject of variation and is not a universal rule for a specific side.

The illness can affect any part of the breast, although breast carcinoma usually starts in the upper and outer quadrants. In this retrospective study, the upper outer quadrant was most commonly involved, accounting for 61.4% of cases. Saha et al. demonstrated that diffuse presentation (45%) was the most common, followed by upper outer quadrant (15.8) involvement [3].

The majority of patients with breast cancer in this retrospective research series (45.7%) appeared at stage II; Rathod et al., in their study, found that stage II (44.5%) and III (44.5%) were the most common stages of presentation [4]. Similarly, Suhani et al. reported stage II as the most typical presentation stage [6]. Nene et al. also concluded that stage II (35.3%) was the most common presentation [7]. All of the aforementioned studies favor our study result. We can conclude from the preceding analysis that stage II is now the most common presentation stage in a developing country, especially in India. The primary reasons for the discrepancy in the stage distribution of breast cancer patients between developing and developed nations have been identified as the absence of screening facilities, protracted medical attention-seeking delays, low socioeconomic status, subpar healthcare systems, and inadequate diagnostic and therapeutic facilities in developing nations.

According to histopathology data in this series, IDC accounted for 85.7% of all cancer cases. Rathod et al. reported IDC as the most common histopathology (85%) among 115 patients [4]. In their study, Suhani et al. reported IDC as the most typical presenting histology [6].

Although the recent trend in the surgical management of breast carcinoma is conservative breast surgery, it is a viable option, especially in the young population. Developing countries like India are now having more concerns among the affected population related to this conservative approach. However, MRM is still a gold standard surgical treatment for breast cancer. In our retrospective analysis, 37 (52.85%) out of 70 patients underwent MRM, which was a standard surgical procedure compared to BCS; Rathod et al. demonstrated that mastectomy was the most common surgical procedure performed (71.3%), with axillary dissection conducted in up to 94.8% of cases [4]. Similarly, Nene et al. demonstrated MRM as a standard surgical procedure (91%) [7].

NACT is now increasing among locally advanced cases. Currently, upfront surgery is limited to early breast cancer and some specific hormonal receptor subtypes (e.g., ER and PR receptor positive) tumors. In this retrospective analysis, 42 (60%) of the 70 patients received NACT. Rathod et al., in their study, showed that 43.5% of the patients received the NACT out of 115 patients [4]. Similarly, Nene et al. showed that 10.3% of patients received NACT [7].

Hormone receptors and HER-2, as well as KI 67% status, are essential in human breast cancer prognosis and management [13]. Although this study did not mention the percentage distribution of different hormone receptor subtypes, the reason for this was the unavailability and readily available facility of hormone receptor study in the specific period. In 70 patients, no one had metastasis at the one-year follow-up.

Within India, the five-year survival rate is 76.3% in localized breast cancer and 47.4% in regional breast cancer, which is much less compared to the West, in which the rates are 89.6% and 75.4%, respectively. A probable reason for the variation in survival rate is the lack of access to the best healthcare services in different countries [14]. We could not have analyzed the survival in our retrospective analysis because we only collected one year of data.

With the advent of chemotherapy and radiotherapy, there has been a paradigm shift in the treatment of breast cancer with the adoption of a “less is more” approach toward surgery [15]. In our study, 47.15% of patients underwent breast conservation, a significant achievement toward the less radical procedure.

There are several limitations of this study. Because this is a retrospective database analysis, we could not get complete data of all the patients. We only analyzed a few patients and did not analyze survival due to limited data collection; however, other Indian centers have reported more extensive series on this.

## Conclusions

Breast cancer is an emerging epidemic in developing countries such as India. The social stigma of carcinoma and hesitancy to show a male doctor, in general, are still challenging problems to address in developing countries. Screening is less effective in developing countries than in developed countries, and thus most cases are presented at an advanced stage. At many levels, a campaign to raise public knowledge of breast self-examination is crucial for early detection of breast cancer and more effective treatment. The clinical spectrum of disease in a country can help identify the disease burden and the presentation of the disease. Individual psychotherapy, family support, group education therapy, and health education are all necessary for patients with cancer. With all of these efforts, the lives of breast cancer patients can be uplifted to a better level.
